# Supplementary material for: Quantifying the Behavioural Relevance of Hippocampal Neurogenesis
Source: PLoS One. 2014 Nov 26;9(11):e113855. doi: 10.1371/journal.pone.0113855 (PMC4245232; doi:10.1371/journal.pone.0113855)
Supplement: Text S1 — Supplementary information. Contains the meta-analysis flow diagram and a discussion of two studies with strong group × neurogenesis interaction effects. (PDF) [file pone.0113855.s001.pdf]

Supporting Information
# Leaps of logic: quantifying the behavioural relevance of hippocampal neurogenesis

Stanley E. Lazic   Johannes Fuss   Peter Gass

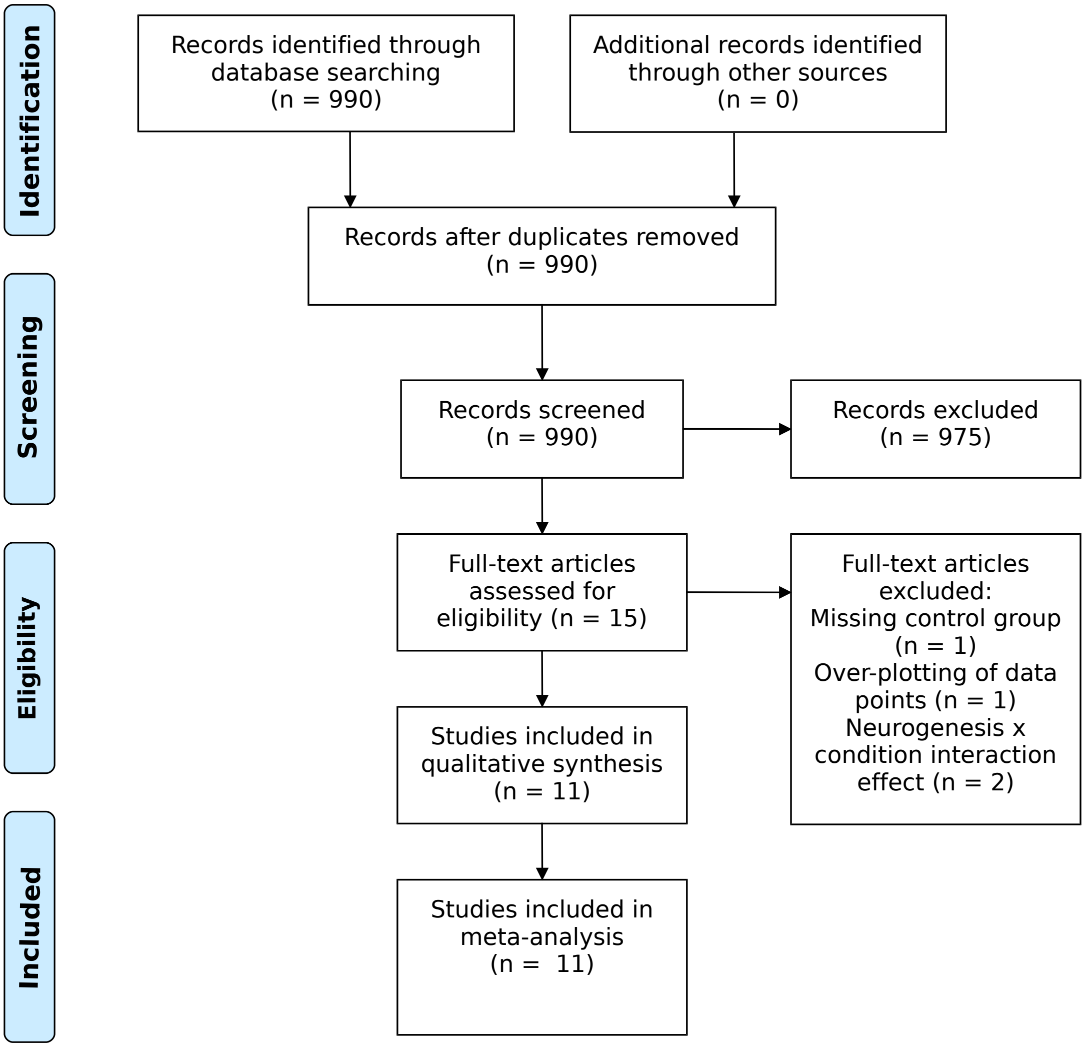


Figure 1: Flow diagram for the systematic review and meta-analysis.

The following two studies by Ishikawa et al. [1] and Drapeau et al. [2] were not included in the combined analysis due to strong interaction effects. While methods exist for estimating such "moderated mediation" models [3], an average effect of the two groups does not provide a reasonable estimate in these cases as the relationship within groups are very different. In addition, it does not fit with the general idea that more neurons are better. The data for the Ishikawa study are presented

in Figure 2, where high values on the behavioural outcome indicate better performance. A positive relationship holds for the memantine group but the opposite is found for the control group. As with many data sets that we re-analysed, the values appeared to form clusters, which might relate to litter-effects or other biological or technical sources of variation. This is evident in the memantine group in the scatterplot of Figure 2. There is one data point with a neurogenesis value below 11, a cluster of three just below 12, and further points near 13. As neurogenesis is on a $\text{Log}_2$ scale in this figure, each whole number represents a doubling of the number of cells between the clusters.

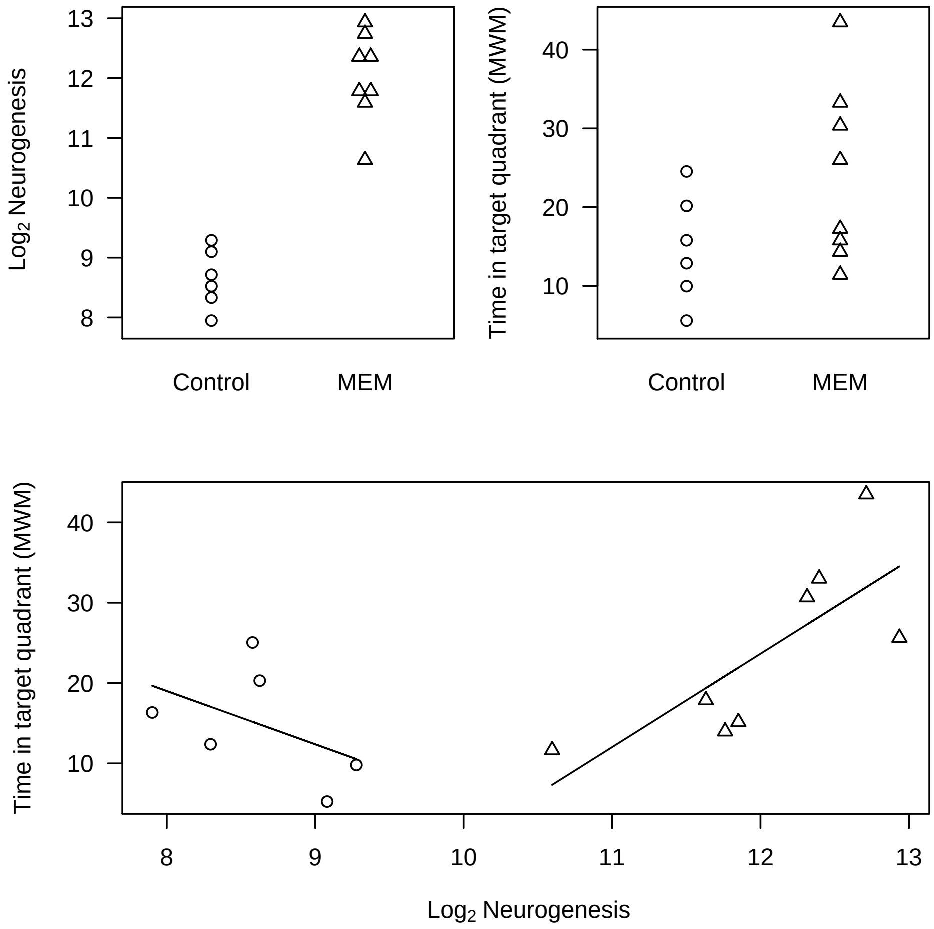


Figure 2: Association between neurogenesis and behaviour differs between groups. MEM = memantine

The results for Drapeau et al. are shown in Figure 3, where the data also appear to form clusters, particularly in the group of young animals. The scatterplot shows that three animals have levels of neurogenesis near 4000, two near 7000, and the rest are in between. In this study lower values on the behavioural outcome indicate better performance. Within the group of older animals, those with high levels of neurogenesis had better performance, but there was no such relationship in the younger group. These results could be explained by a floor-effect, where values near 500 on the behavioural outcome are the best that can be achieved and thus the neurogenesis-behaviour relationship no longer holds once this performance level is reached. While plausible, this pattern was not found in the other studies and indeed is the opposite of the Ishikawa study above, where the behaviour-neurogenesis relaionship was strongest in the high-neurogenesis group.

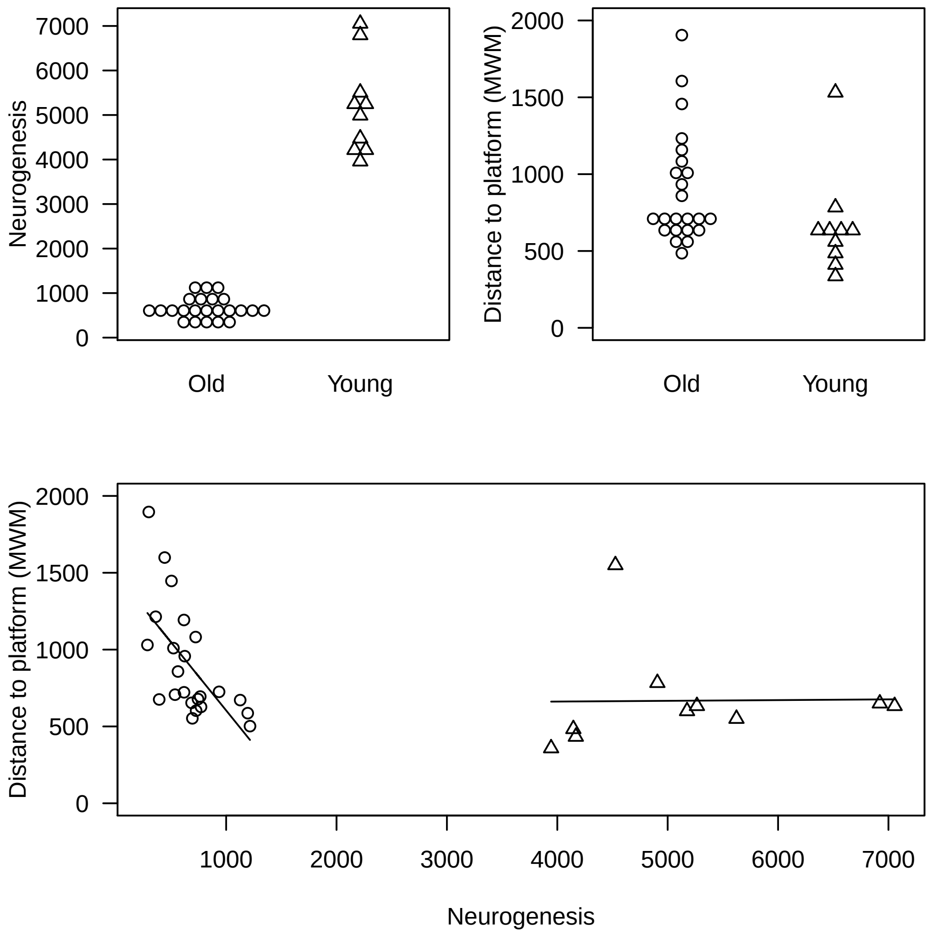


Figure 3: Association between neurogenesis and behaviour differs between groups.

# References


[1] Ishikawa R, Kim R, Namba T, Kohsaka S, Uchino S, et al. (2014) Time-dependent enhancement of hippocampus-dependent memory after treatment with memantine: Implications for enhanced hippocampal adult neurogenesis. Hippocampus .

[2] Drapeau E, Mayo W, Aurousseau C, Moal ML, Piazza PV, et al. (2003) Spatial memory performances of aged rats in the water maze predict levels of hippocampal neurogenesis. Proc Natl Acad Sci U S A 100: 14385–14390.

[3] Preacher KJ, Rucker DD, Hayes AF (2007) Addressing moderated mediation hypotheses: theory, methods, and prescriptions. Multivariate Behavioral Research 42: 185–227.
